# Donkey Epididymal Transport for Semen Cooling and Freezing

**DOI:** 10.3390/ani10122209

**Published:** 2020-11-25

**Authors:** Yamilka Lago-Alvarez, Giorgia Podico, Lorenzo G. Segabinazzi, Lais L. Cunha, Leonardo Barbosa, Carolyn E. Arnold, Fabio S. Lima, Luise T. King, Amy K. McLean, Igor F. Canisso

**Affiliations:** 1 Department of Veterinary Clinical Medicine, College of Veterinary Medicine, University of Illinois, Urbana, IL 61801, USA; yla3@cornell.edu (Y.L.-A.); gpodico@illinois.edu (G.P.); lgseg@hotmail.com (L.G.S.); laisl@illinois.edu (L.L.C.); lbarbosa@illinois.edu (L.B.); falima@ucdavis.edu (F.S.L.); 2 Department of Large Animal Clinical Sciences, College of Veterinary Medicine and Biomedical Sciences, Texas A&M University, College Station, TX 77840, USA; cearnold@tamu.edu; 3 Department of Veterinary Clinical Medicine, University of Arizona, Oro Valley, AZ 85704, USA; tluiseking@arizona.edu; 4 Department of Animal Sciences, University of California, Davis, CA 95161, USA; acmclean@ucdavis.edu

**Keywords:** epididymis, cooled-shipped epididymides, castration, testis, semen cryopreservation

## Abstract

**Simple Summary:**

In the event of death, euthanasia, or forceful castration for medical reasons, epididymal semen harvesting represents the last opportunity to preserve the genetic material of valuable sires. However, this technique has yet to be tested in donkeys. Three experiments were carried out to assess epididymal semen cooling and freezing in donkeys. In experiment 1, semen cooling and freezing were conducted immediately after castration, and in experiments 2 and 3, epididymides were shipped overnight, and then epididymal semen cooled and frozen. Results showed that cooling of epididymal semen up to 24 h after harvesting did not affect motility parameters or plasma membrane integrity. Collectively, the post-thaw results revealed low motility parameters across groups; At the same time, the plasma membrane integrity did not reflect this trend, and the values remained high, suggesting that there was a lack of epididymal sperm activation after freezing. In summary, freshly harvested and cooled-shipped epididymal donkey semen had satisfactory semen parameters. New studies need to address donkey epididymal semen fertility in mares and jennies.

**Abstract:**

The objectives of this study were to assess the cooling and freezing of donkey epididymal semen harvested immediately after castration (Experiment 1, *n* = 4) or after the shipment (24 or 48 h) of epididymides attached to testicles (Experiment 2, *n* = 14) or dissected apart (Experiment 3, *n* = 36). In each experiment, semen was frozen immediately (Non-Centrif) in an egg yolk-based semen extender (EY) or after processing through cushion-centrifugation (Centrif) while extended in a skim milk-based extender (SC). In all three experiments, cooled, pre-freeze, and post-thaw epididymal semen was assessed for total motility (TM), progressive motility (PM), plasma membrane integrity (PMI), and high mitochondrial membrane potential (HMMP). Data were analyzed with R using mixed models and Tukey’s test as posthoc. Results showed that the cooling of epididymal semen up to 24 h after harvesting did not affect motility parameters or plasma membrane integrity; furthermore, in Experiment 3, the post-thaw evaluation of both Centrif and Non-Centrif achieved similar TM and PM. Collectively, the post-thaw results revealed low motility parameters across groups; while, the PMI and HMMP did not reflect this trend, and the values remained high, suggesting that there was a lack of epididymal sperm activation with either centrifugation or extenders. In summary, freshly harvested and cooled-shipped and cooled semen had satisfactory semen parameters. Future studies need to address donkey epididymal semen fertility in mares and jennies.

## 1. Introduction

In the American continent, donkey-jacks (jacks, Equus asinus) are primarily used for breeding mares (Equus caballus) to produce mules (Equus mulus) [1,2]. Top mule producers are increasingly prized animals in the donkey and mule show industry in the United States and Brazil [1,3]. Most recently, there has been a massive increase in the number of breeding programs to produce donkey-hide gelatin (ejiao, colla cori asini), a collagen product extracted from the skin, which is the central pillar of traditional Chinese medicine [2]. The increased value of show mule sires and interest for donkeys to produce ejiao has generated an exponential interest in donkey semen freezing [2]. 

While much progress has been made in recent years to cryopreserve ejaculated jack semen [2,4,5], harvesting epididymal semen has yet to be investigated in this species. In the event of death, euthanasia, or forceful castration for medical reasons (e.g., trauma to the scrotum, life-threatening conditions, and testicular neoplasia), epididymal semen harvesting represents the last opportunity to preserve the genetic material of valuable sires [6]. Since most jacks are located far away from laboratories capable of harvesting and freezing epididymal semen, cooling and shipping the epididymides alone or attached to testicles could prove advantageous. In horses, epididymal shipment for semen freezing has been successfully carried out by different groups [7,8,9,10]; however, to our knowledge, this approach has not been investigated in donkeys.

Furthermore, the preservation of epididymal spermatozoa could be a valuable tool to preserve the genetics of endangered domestic donkey breeds (e.g., Amiata, Catalan, Andalusian, Baudet Du Poitou, and Martina Franca) and endangered wild donkey species, such as the African wild ass (Equus africanus), Somali wild ass (Equus africanus somaliensis), and Asiatic wild ass (Equus hemionus) [11,12]. To prevent the imminent extinction of these animals, decisive conservation measures are needed to preserve the genetic material of endangered breeds and species [12]. Historically, routine semen collections of captive equids in zoological collections have been performed via electroejaculation under chemical restraint [12]. However, when ejaculated sperm are not available due to death or euthanasia for medical reasons, epididymal semen harvesting could be used as an alternative method. Despite having valuable species in the collection, zoos across the world are often not equipped or do not have the expertise to process epididymal semen for cryopreservation. Therefore, zoos could benefit from shipping epididymides to referral centers acquainted with freezing epididymal semen.

Numerous harvesting methods of epididymal semen have been evaluated and optimized in the domesticated species [8,13,14,15,16]. In horses, the methods available for epididymal semen harvesting include direct aspiration, slicing, and flotation, or retrograde flushing of the cauda epididymis; [8,17]. Although there is no consensus regarding the most suitable method, the latter appears to be the most widely used [10]. Furthermore, if the retrograde flushing is performed with a freezing extender, the semen can be directly frozen and requires no further processing; however, if the retrograde flushing is performed with a cooling extender, the harvested semen needs to be centrifuged, the supernatant discarded, and the pellet resuspended in a semen freezing extender. While some authors advocate for the direct method [8], others advocate for the other approach, considering centrifugation a necessary step to activate epididymal sperm motility [18,19]. The only study comparing the two approaches failed to find any differences in post-thaw semen parameters for stallion epididymal semen processed via either approach [6]. The use of centrifugation is controversial in harvested epididymal semen; since epididymal sperm usually yields high sperm concentration, there is no need to concentrate sperm and remove seminal plasma [6]. In the stallion, semen centrifugation can lead to a sperm loss from 10 to 45% [20,21]. Therefore, the objectives of the present study were to compare the cooling and freezing ability of donkey epididymal semen obtained either from retrograde flushing and direct freezing or retrograde flushing and then cooling and freezing after cushion centrifugation. In addition, this study aimed to assess donkey epididymal semen cooling and freezing harvested immediately after castration and on cooled shipped epididymides attached to testicles or dissected apart during transport. We hypothesized that the semen quality parameters of donkey epididymal sperm result in equivalent post-cooling and post-thaw semen parameters irrespective of the method used for sperm harvesting, processing, and storage time.

## 2. Materials and Methods

Three experiments were conducted in the present study, from October 2019 to February 2020. The donkey epididymal semen was harvested immediately after castration (Experiment 1) or after the epididymides were shipped (Experiments 2 and 3). In Experiment 2, epididymides attached to the testicles were shipped in eight passive semen cooling containers (Equitainer II; Hamilton Research, Inc., Ipswich, MA, USA). In Experiment 3, epididymides dissected away from the testes were shipped in twelve passive cooling containers (Botuflex, Botupharma USA, Phoenix, AZ, USA). In Experiments 2 and 3, subsets of the passive cooling containers were randomly chosen and processed after arrival (cooled-shipped 24 h, *n* = 8 and *n* = 20 pairs for Experiments 2 and 3, respectively) and the next day (cooled-shipped 48 h, *n* = 6 and *n* = 16 pairs for Experiments 2 and 3, respectively).

### 2.1. Experiment 1: On-Site Epididymal Semen Cooling and Freezing

#### 2.1.1. Animals and Castration

Four privately-owned jacks (2 American Mammoth and 2 Miniature, ranging from 2 to 10 years old, bodyweights in the range of 95–350 kg) were enrolled in this study. The jacks belonged to four different clients and were presented for routine castration at the University of Illinois Veterinary Teaching Hospital, Urbana, IL (40.1206°N, 88.2073°W). The owners reported that their diets consisted of mixed alfalfa–grass hay and no grain supplementation. This experiment was carried out to determine whether the techniques used to process horse epididymal semen could be effectively applied to donkeys. For each jack, a full physical examination including palpation of the scrotal content was performed prior to castration to exclude gross scrotal abnormalities (e.g., hernias, no adhesions, or lacerations and confirmation of two descending testicles).

Standard intravenous general anesthesia was induced with triple-drip, which includes xylazine, midazolam, and ketamine [22]. Once under general anesthesia, routinely closed castration was performed through a scrotal approach using Reimer’s emasculators as previously described [22]. In addition, for each testicle, two mosquito hemostats were placed at the vas deferens distal to the emasculator to minimize the loss of semen. The cord was transected immediately distal to the emasculator and proximal to the mosquito hemostats, removing the testicle and a portion of the spermatic cord. Client consent was obtained from all four owners.

#### 2.1.2. Epididymal Semen Harvesting

The epididymides were rinsed with room temperature sterile Lactated Ringer’s Solution and wiped with cotton gauze to remove any contamination from blood or debris. The epididymides were dissected from the testes, as previously described [6]. Briefly, the vas deferens and tail of the epididymides were dissected free of blood vessels and connective tissue working distally from the vas deferens toward the body of the epididymis. Then, the tail of the epididymis was transected with a straight-bladed Mayo scissor. Each epididymal tail was weighed on a scale before further processing. An intravenous catheter (16 Ga × 13 cm, MilaCath^®^) was inserted into the proximal end of the ductus deferens and slowly flushed with 5–10 mL of a cooling extender based on sodium caseinate cyclodextrin–cholesterol loaded (SC; BotuSemen Gold, Botupharma USA, Phoenix, AZ, USA) or egg yolk-based semen freezing extender (EY; Botucrio, Botupharma USA) as previously described [6]. One epididymis of each pair was submitted to retrograde flushing with EY, while the contralateral epididymis was flushed with SC. Once the tail of the epididymis was fully distended, it was cut at its most distal portion with a straight-bladed Mayo scissor, and the fluid containing the semen allowed to outflow into a 50 mL conical tube (Thermo Scientific™, Nunc™, Rochester, NY, USA) [6].

#### 2.1.3. Epididymal Semen Processing

The semen recovered from the epididymides flushed with the EY extender was further extended at 100 million sperm/mL, and a portion was frozen immediately without centrifugation (Non-Centrif-0 h) (as described in Section 2.1.4) or packed into an 18 oz disposable plastic bag (Whirl-Pak^®^, Nasco Inc., Fort Atkinson, WI, USA) and maintained in a passive cooling semen container (BotuFlex, Botupharma USA) for 24 h before being frozen in a similar manner (Non-Centrif-24 h). The contralateral epididymides flushed with SC extender were extended at 100 million sperm/mL and stored in a passive cooling container (BotuFlex) for 24 h (Centrif-24 h), or cushion-centrifuged and then re-extended with the same extender and then stored in the same container for 24 h (C-Centrif-24 h) or resuspended in the EY extender (Centrif-0 h) and frozen. Cushion centrifugation was performed as previously described [6]. Briefly, the extended semen was loaded in 50 mL conical tubes (Thermo Scientific™, Nunc™) with the addition of 1 mL cushion fluid (Red-Cushion, Botupharma USA) placed at the bottom of the tube with a blunted spinal needle (18 Ga × 13.5 cm). Centrifugation was performed at 1000× *g* × 20 min at room temperature.

Following centrifugation, the supernatant and cushion solution was discarded. The concentration of the remaining pellet was assessed as described below, and the semen cushion-centrifuged was either re-extended in the same SC extender at 100 million sperm/mL or in the EY extender at 100 million sperm/mL. The semen extended in the EY extender was frozen immediately. After 24 h of cooled storage, semen was cushion-centrifuged, resuspended in the EY extender, and then frozen. Sperm motility parameters, plasma membrane integrity (PMI), and high-mitochondrial membrane potential (HMMP) were assessed immediately before (pre-cooling) and after 24 h (post-cooling) of cooled-storage in a passive cooling semen container, and before and after cushion centrifugation, as described in Section 2.4 and Section 2.5.

#### 2.1.4. Epididymal Semen Freezing

Once semen was finally extended with the EY extender at 100 million sperm/mL, it was manually loaded in 0.5 mL straws and sealed with a portable straw sealer (UltraSeal21TM, Minitube of America, Vernon, WI, USA). Once the straws were sealed, they were placed in a cold room at 5 °C for 20 min. The isothermal box (Lifoam™, Fishers, IN, USA) of 42 L capacity was filled with a depth of 6 cm of liquid nitrogen. The straws were placed horizontally on a rack at 3 cm above the liquid nitrogen for 20 min in the airtight isothermal box. Subsequently, the straws were immersed in liquid nitrogen [8]. Then, the straws were loaded into canes and transferred to liquid nitrogen tanks until further analyses. The thawing of semen was performed by placing one straw at the time in a water bath at 38 °C for 60 s. Then, the samples were assessed for either treatment to determine the sperm motility parameters, PMI, and HMMP, as described in Section 2.4 and Section 2.5.

### 2.2. Experiment 2. Cooled-Transported Testicles and Epididymides for Semen Cooling and Freezing

#### 2.2.1. Animals and Castration

Fourteen feral small standard jacks (ranging from 4 to 13 years old, bodyweights in the range of 150–200 kg) maintained in corrals at the Bureau of Land Management facility in Florence, AZ (33.0315° N, 111.3873° W) were enrolled in this experiment. The agency gave us permission to use the samples harvested from these animals. This experiment was conducted to determine whether harvesting the testicles and epididymides under field conditions and shipping them to a specialized laboratory could be an alternative approach to cryopreserve epididymal donkey semen. The donkeys were housed in small groups and fed alfalfa and bermudagrass hay. All jacks were gathered at least six months from the states of Arizona, Nevada, and California. Physical examination was performed, which included the confirmation of two descending testicles with no adhesions, abrasions, or lacerations, and only jacks with unremarkable physical examinations were enrolled in the study.

All jacks were anesthetized with the intravenous administration of detomidine, butorphanol, and ketamine [22]. Once under general anesthesia, open castration was performed using Henderson’s instrument, as previously described [22]. For each testicle, ligations were placed at the vas deferens immediately after the cord transection. The testes attached to the epididymis and spermatic cord were maintained in an isothermal box for about one hour until processing for shipping.

#### 2.2.2. Epididymal Processing and Shipping

The epididymides attached to the testicles were rinsed and packed in a disposable, plastic Whirl-Pak^®^ bag containing fresh skim-milk based extender (25 mL, Botusemen, Botupharma USA, Phoenix, AZ, USA), and shipped overnight in a passive cooling device (Equitainer II; Hamilton Research, Inc.) to the University of Illinois Veterinary Teaching Hospital, Urbana, IL, USA for semen cooling and freezing. The Equitainer cans were deep-frozen at −20 °C for at least 24 h. Upon arrival, each Equitainer contained one to two pairs of testicles with the epididymides attached, individually packed in Whirl-Pak^®^ without an isothermolizer cup.

#### 2.2.3. Epidydimal Semen Processing, Cooling, and Freezing

The samples (*n* = 14 pairs) were randomly divided and either processed after arrival (Cooled-shipped 24 h, *n* = 8) or the next day (Cooled-shipped 48 h, *n* = 6). Once each passive cooling device was opened, the extender’s temperature where the sample was submerged was assessed. The epididymides were dissected from the testes as previously described [6]. Briefly, the epididymides were rinsed with room temperature sterile Lactated Ringer’s Solution and wiped with cotton gauze to remove any contamination from blood or debris. Each epididymal tail was weighed before processing. Thereafter, retrograde epididymal flushing was performed as aforementioned in Experiment 1—Section 2.1.2. The lumen of the ductus deferens was cannulated with a 16 Ga × 13 cm (MilaCath^®^) and flushed with 5–10 mL of the SC or EY extenders. Further semen processing and freezing were performed as described in Experiment 1—Section 2.1.3 and Section 2.1.4. The assessment of sperm motility parameters, PMI and HMMP, was performed for all samples before (pre-cooling) and after cooling (post-cooling) and pre-freezing and post-thaw, as described in Section 2.4 and Section 2.5.

### 2.3. Experiment 3: Cooled-Transported Epididymides for Semen Cooling and Freezing

#### 2.3.1. Animals and Castration

Thirty-six feral small standard jacks (ranging from 2 to 17 years old, 100–300 kg in body weight) were housed in a 172-acre ranch in San Angelo, TX, USA (31.4638°N, 100.4370°W) at the Peaceful Valley Donkey Rescue. This experiment was performed to determine the feasibility of dissecting the epididymides away from the testicles and shipping overnight in a passive cooling container to a referral laboratory equipped to perform semen cryopreservation and to investigate the epididymal semen harvesting and processing techniques with a larger number of animals. The jacks were maintained in a herd of approximately 1000 donkeys, and their diet consisted of free access to coastal bermudagrass, Sudan grass hay, and the grain supplementation of ADM^®^ Sweet UniqueTM 14% (ADM Animal Nutrition™, Quincy, IL, USA). The rescue facility granted us full access to the samples and records to conduct the present study. All jacks were gathered from Goldstone, Fort Irwin, and Butte Valley in California. Only jacks with unremarkable physical examinations were enrolled in this study.

Each donkey was placed under general anesthesia by administering intravenous xylazine and ketamine [22]. Prior to the aseptic preparation of the surgical site, both testicles were evaluated for gross abnormalities. Following anesthetic induction and aseptic preparation of the scrotum, each donkey was castrated with an open technique using a Serra and/or Reimer emasculators as previously described [22]. For each testicle, ligation of the vas deferens was performed while maintaining the longest portion to prevent loss of semen from the epididymis and deferent duct before the spermatic cord was transected. The samples were maintained in an isothermal box until processing within one hour of surgical removal.

#### 2.3.2. Epididymal Processing and Shipping

The epididymides were dissected away from the testes and packed in a disposable, plastic Whirl-Pak^®^ bag containing fresh skim-milk based extender (25 mL, Botusemen), and cooled-shipped in a passive cooling device overnight (Botuflex). The Botuflex consists of two ice packs placed on each side of the device. Ice packs were deep-frozen at −20 °C for 24 h. Three pairs of epididymides individually packed in disposable plastic bags (Whirl-Pak^®^) were placed in the well of the container.

#### 2.3.3. Epidydimal Semen Processing, Cooling, and Freezing

Samples were randomly processed after arrival (cooled-shipped 24 h *n* = 20 pairs) or in the next day (Cooled-shipped 48 h *n* = 16 pairs). The epididymides were rinsed with Lactated Ringer’s Solution at room temperature and wiped with cotton gauze to remove any contamination from blood or debris. Each epididymal tail was weighed before processing. Thereafter, retrograde epididymal flushing was performed as aforementioned in Experiment 1—Section 2.1.2. The lumen of the ductus deferens was cannulated with a 16 Ga × 13 cm (MilaCath^®^) and flushed with 5–10 mL of the SC extender or EY extenders. Further semen processing and freezing were performed as described in Experiment 1—Section 2.1.3 and Section 2.1.4. The assessment of sperm motility parameters, PMI and HMMP was performed for all samples before and after cooling and freezing as described in Section 2.4 and Section 2.5.

### 2.4. Assessment of Sperm Concentration and Motility

Across the three experiments, sperm concentration was determined using an automated cell counter (Nucleocounter^®^ SP-100™, Chemometec, Denmark) following the manufacturer’s instructions. Briefly, 50 μL of semen was diluted in 5 mL of lysis buffer (Reagent S100, Chemometec, Denmark) and loaded into the cassettes before the assessment.

Throughout the three experiments, the assessment of the sperm motility parameters was performed using computer-assisted sperm analysis (CASA) with default settings recommended by the manufacturer (Spermvision, Minitube of America, Verona, WI, USA) for equine sperm. The preset values for the CASA were: static cell area 14–100 µm^2^, straightness threshold for progressive motility 90%, average path velocity threshold for static cell <9.5 μm/s, light-emitting diode illumination intensity 180–255. Each sample was incubated for 10 min at 38 °C before each evaluation. A small aliquot (10 µL) of extended semen was placed on a pre-heated slide with a coverslip for the assessments. Motility parameters assessed included the total percent of sperm motility (TM), progressive sperm motility (PM), curvilinear velocity (VCL, μm/s), average path velocity (VAP, μm/s), and straight-line velocity (VSL, μm/s).

### 2.5. Flow Cytometry Analysis

The evaluation of PMI and HMMP was conducted using a spectral flow cytometer, as previously described [23]. Briefly, the staining solution of Zombie Green dye (#423112 Biolegend, San Diego, CA, USA) was freshly prepared with 100 μL of DMSO added to each vial of dye; similarly, MitoTracker Deep Red FM (M22426, Molecular Probes, Eugene, OR, USA) stock solution was prepared by adding DMSO to create a 10 μM solution. The stock solution was aliquoted and frozen at −20 °C until it was used. 

One milliliter containing 50 million sperm/mL was centrifuged (600× *g* × 10 min) and then resuspended in PBS to a concentration of 3–5 million sperm/mL. Subsequently, a 100 μL aliquot of this solution was stained with both dyes (1 μL of Zombie Green and 1 μL MitoTracker Deep Red). After mixing, the sample was incubated for 30 min at room temperature in the dark. The incubation was followed by centrifugation (400× *g* × 5 min). The supernatant was discarded, and each pellet was fixed with 500 μL of 2% buffered formalin and stored in the dark until flow cytometric evaluation. Before the flow cytometric analysis, samples were washed with 1 mL of PBS, centrifuged at 400× *g* × 5 min, and resuspended in PBS (250 μL). The analyses of the stained samples were conducted using a full-spectrum detector based (filter-less) Cytek Aurora Flow Cytometer (Cytek Biosciences Inc., Fremont, CA, USA). The analysis was concluded when at least 10,000 fluorescent gated events were recorded. Zombie Green was excited and detected with a 488 nm fluorescence detector, whereas MitoTracker Deep Red was excited with a 644/665 nm detector. Unstained and single-stained controls were used to unmix the signals. As previously described [23], four subpopulations of sperm were identified. The populations of sperm with intact (low Zombie Green signal) or damaged (high Zombie Green signal) plasma membrane were subdivided into low or high mitochondrial membrane potential based on the intensity of the signal given by Mitotracker Deep Red staining. Debris was manually excluded based on the minimal emitted fluorescence. Data from the flow cytometer were exported and analyzed with FlowJo (FlowJo v. 10 Software, Ashland, OR, USA). The PMI and HMMP potential were accounted for comparisons across groups.

### 2.6. Statistical Analysis

Assumptions of linearity, homogeneity of variance, and normality of the residuals were tested with plots, Levene’s, and Shapiro–Wilk test. Data analyses were carried out with RStudio v 3.2.3 (RStudio Team, Boston, MA). Data were analyzed by mixed models with cooled-storage, shipment duration, semen freezing, and the type of extender considered as fixed effects and the individual donkey as a random effect. Tukey’s test was used for post-hoc comparisons. Statistical significance was set at *p* < 0.05. A statistically significant tendency was determined with 0.05 < *p* < 0.1. All data are presented as mean ± SEM.

## 3. Results

### 3.1. Experiment 1. On-Site Epididymal Semen Cooling and Freezing

The retrograde flushing of the epididymal tails was successfully performed on all four pairs. None of the epididymides had appreciable gross lesions. The total number of sperm recovered from each pair was 9.6 ± 2.2 billion (range 5.1–18.1 billion) (Supplementary Materials Table S1). The average weight of each epididymis was 16.7 ± 2.5 g (range 10.1–25.1 g) (Supplementary Materials Table S1).

#### 3.1.1. Cooling

There were no differences in any of the parameters (TM, PM, VAP, VCL, VSL, PMI, and HMMP) measured at any timepoints during cooling or between groups (Table 1) (*p* > 0.05). The progressive motility of the donkey semen harvested in the EY extender immediately after castration tended (*p* = 0.08) to yield greater values compared to the semen recovered in the SC extender (Table 1).

#### 3.1.2. Freezing–Thawing

There was a reduction in post-thaw TM and PM in comparison with pre-freeze values for all groups (*p* < 0.05) (Table 2). Groups Non-Centrif-24 h and Centrif-0 h had a reduction in post-thaw PMI in comparison to the pre-freeze values (*p* < 0.05) (Table 2). Post-thaw HMMP was reduced in comparison to the pre-freeze values for the Non-Centrif-0 h group (*p* < 0.05) but not different for the remaining groups (*p* > 0.05) (Table 2). There were no differences between the groups for the post-thaw results of TM, PM, PMI, and HMMP (*p* > 0.05) (Table 2). Post-thaw, there was a reduction in VAP, VCL, and VSL in comparison to the pre-freeze values for the groups Non-Centrif-0 h and Centrif-0 h (*p* < 0.05) (Table 2). Pre-freeze values of VCL, VSL, and VAP were greater at 0 h than the ones at 24 h groups (Non-Centrif-0 h vs. Non-Centrif-24 h and Centrif-0 h and Centrif-24 h) (*p* < 0.05) (Table 2).

### 3.2. Experiment 2. Cooled-Transported Testicles and Epididymides for Semen Cooling and Freezing

The total number of sperm recovered was 6.4 ± 0.7 billion (range 0.4–16.2 billion) (Supplementary Materials Table S2). One donkey was excluded from the study due to insufficient sperm counts to assess all the different endpoints. The average weight for each epididymis was 25.1 ± 2.0 g (range 10.8–56.7 g) (Supplementary Materials Table S2). None of the epididymides had gross morphological abnormalities. The average temperature of the samples upon arrival was 7.6 ± 0.7 °C (range 5–9.7 °C, cooled-shipped 24 h), and 15.5 ± 0.4 °C (range 14–17 °C, cooled-shipped 48 h).

#### 3.2.1. Cooling

There were no differences across groups for any of the endpoints (TM, PM, PMI, HMMP, VCL, VSL, and VAP) assessed pre- and post-cooling or between samples allocated to the cooled-shipped 24 h and the cooled-shipped 48 h (*p* > 0.05) (Table 3).

#### 3.2.2. Freezing–Thawing

There were no differences between the cooled-shipped 24 h vs. the cooled-shipped 48 h (*p* > 0.05) (Table 4). Post-thaw, there was an overall reduction in TM and PM across the groups in comparison to the pre-freeze values (Table 4) (*p* < 0.05). However, there were no differences between groups for pre-freeze and post-thaw TM and PM (*p* > 0.05). Pre- and post-freezing VCL and VSL did not differ across groups (*p* > 0.05) (Table 4). Post-freezing VAP decreased in comparison to pre-freeze for the Non-Centrif-24 h group (*p* < 0.05), but it was not different for other groups (*p* > 0.05) (Table 4). There was a reduction of HMMP in post-thaw samples in comparison to pre-freeze values for group Centrif-24 h (*p* < 0.05), but it was not different between other groups (*p* > 0.05) (Table 4).

### 3.3. Experiment 3. Cooled-Transported Epididymides Tails for Semen Cooling and Freezing

The retrograde flushing of the epididymis tail was successfully performed on all thirty-six pairs; total sperm count average was 8.4 ± 0.3 billion (range 0.4–19 billion) from each epididymis. The average weight of each epididymis was 28.5 ± 0.6 g (range 20.8–45 g) (Supplementary Materials Table S3). The average temperature of the samples upon arrival was 9.4 ± 0.5 °C (range 7–13 °C, cooled-shipped 24 h), and 17.2 ± 0.5 °C (range 14–20 °C, cooled-shipped 48 h).

#### 3.3.1. Cooling

Semen harvested in the EY extender had greater TM and PM than SC and C-SC for both cooled-shipped 24 h and cooled-shipped 48 h (*p* < 0.05) (Table 5). It is noteworthy that PM in the EY group increased after 24 h of cooling in a passive-cooling device for both cooled-shipped 24 h and cooled-shipped 48 h (*p* < 0.05) (Table 5).

There were no differences in PMI for any of the groups for cooled-shipped 24 h (*p* > 0.05) (Table 5). In the cooled-shipped 48 h, PMI increased after cooling in comparison to pre-cooling for EY (*p* < 0.05). In addition, after centrifugation (SC), the PMI was lower in cooled-shipped 48 h than in the cooled-shipped 24 h (*p* = 0.0006) (Table 5). There were no differences between groups for VAP and VSL across timepoints and processing (*p* > 0.05), whereas the VCL was higher in the EY semen than in the SC semen cooled-shipped for 24 h (*p* < 0.05) (Table 5).

#### 3.3.2. Freezing–Thawing

There was a reduction in post-thaw TM and PM across groups and cooled-shipped 24 h and cooled-shipped 48 h (*p* < 0.05) (Table 6). In cooled-shipped 24 h, the pre-freeze values of PM in Non-Centrif-24 h were greater than the one in Non-Centrif-0 h (*p* < 0.05) (Table 6). The pre-freeze values of PM in Non-Centrif-24 h was greater than those in Centrif-24 h in cooled-shipped 24 h (*p* < 0.05) (Table 6).

Post-thaw velocity parameters (VSL, VAP, VCL) were reduced in cooled-shipped 24 h across groups in comparison to the pre-freeze values (*p* < 0.05), except for group C-Centrif-24 h which maintained its pre-freeze values (*p* > 0.05) (Table 6). Pre-freezing values of VSL and VCL were greater in Centrif-0 h than in Centrif-24 h (*p* < 0.05). Post-thaw PMI decreased in comparison to pre-freeze values for all groups (*p* < 0.05), except for semen in C-Centrif-24 h group, which maintained its pre-freezing values (*p* > 0.05) (Table 6). The population of sperm HMMP did not change from pre-freeze to post-thaw (*p* > 0.05) (Table 6). Pre-freeze HMMP was higher in Non-Centrif-0 h than semen in Centrif-0 h (*p* < 0.05) (Table 6). Moreover, cooled-shipped 24 h pre-freezing values of semen extended in Centrif-0 h group had less sperm with HMMP than any of the other groups at 24 h (*p* < 0.05) (Table 6).

Cooled-shipped 48 h pre-freeze values of TM and PM were greater in Non-Centrif-24 h than in Centrif-24 h (*p* < 0.05). Post-thaw VSL and VAP decreased in comparison with pre-freeze values in groups Non-Centrif-0 h, Centrif-0 h, and Non-Centrif-24 h. Moreover, post-thaw VCL decreased in group Non-Centrif-24 h in comparison with pre-freeze values (*p* < 0.05) (Table 6). Pre-freeze values of VCL and VAP were greater in the Centrif-0 h group than groups Centrif-24 h and C-Centrif-24 h (*p* < 0.05). The values of PMI pre-freeze of semen extended in Non-Centrif-24 h was greater than in Non-Centrif-0 h (*p* < 0.05) (Table 6). Post-thaw HMMP did not change after freezing with any extender or methods or processing (*p* > 0.05) (Table 6).

## 4. Discussion

The present study was designed to assess donkey epididymal semen cooling and freezing, from freshly harvested and cooled-shipped specimens. The first experiment was conducted to determine whether freshly castrated donkeys could have epididymal semen harvested, cooled, and then frozen. The second experiment was conducted to determine the feasibility of collecting, packaging, and shipping testes attached to the epididymis and spermatic cord to a referral laboratory for epididymal semen harvesting, cooling, and freezing. This design was implemented to simulate a scenario when a layperson or an inexperienced practitioner was unable to effectively dissect the epididymides from the testes before shipping to a referral laboratory. Additionally, a subset of the shipment was processed after delivery (cooled-shipped 24 h), and another subset was processed the next day (cooled-shipped 48 h). The delay in processing the samples was created to mimic common problems encountered in clinical practice. It has been estimated that about 5% of the semen shipments in the United States may be delayed for various reasons. Furthermore, animals located in rural and remote indigenous locations cannot have their testes and/or epididymis timely shipped to a referral laboratory for further processing. Experiment three was conducted with a larger sample size to determine the feasibility of dissecting the epididymal tail, packaging, shipping, and further processing for cooling and freezing. In addition, shipping the epididymides dissected from the testes minimizes the potential for blood contamination and debris, decreases the amount of space occupied in a passive cooling container and shipping charges. In this experiment, a similar approach for delayed processing (cooled-shipped 24 h vs. 48 h) was performed.

In all three experiments, the concept of cooling donkey semen after harvesting was assessed to simulate cooled-shipped semen for potential artificial insemination with cooled semen or as a means to pre-process semen before freezing. Cooling epididymal semen after harvesting could be a useful strategy if a popular jack needs to be castrated or euthanized in the middle of the breeding season when there are mares or jennies lined up to be inseminated. Breeding mares or jennies with cooled semen could circumvent the problems with frozen semen (e.g., frequent follicular checks, low fertility, and excessive post-breeding endometrial inflammation) [24]. Interestingly, the cooling extender used in the present study contains cholesterol-loaded cyclodextrin (CLC). Although this compound had not been tested yet to cool donkey epididymal semen, satisfactory fertility rates have been obtained in mares inseminated with donkey ejaculated semen extended in the CLC SC extender (Papa FO, personal communication). In addition, CLC has been reported to improve the sperm viability of post-thawed donkey semen with no detrimental effect on fertility rates in mares [25]. The addition of CLC improved semen quality and fertility of cooled stallion semen [25,26]. The CLC has also been reported in one study to decrease cooling-induced damages and to improve the semen freezing ability of stallion’s epididymal sperm [27].

Cooling epididymal semen before freezing was assessed as a strategy herein; the authors were interested in answering whether donkey semen could be cooled overnight before being frozen the next day. Such a strategy could prove to be useful in clinical practice. For instance, if a donkey needs to have epididymal semen harvested and processed outside regular working hours, the findings of the present study suggest that semen can be harvested, cooled, and frozen the next day. Some mature jacks have a large amount of semen, which requires a long processing time. The jacks used in the present study were in general, of the small frame (except two among the young animals used in Experiment 1); however, some yielded close to 40 billion sperm. Larger breeds such as Mammoth, Poitou, and Catalan can easily surpass that amount [2]. While the fertility of this type of semen has not been assessed, the present study provided an introductory concept for its use and future studies.

Across experiments, epididymal semen cooling displayed satisfactory parameters, and semen flushed with EY tended to yield superior results than semen flushed with SC. The use of a freezing extender for cooling semen may come as a surprise to investigators as it has been proposed that stallion and donkey semen are sensitive to glycerol and high concentrations of this cryoprotectant affect the fertility of semen extended in milk-based or milk-based protein extenders [28]. Vidament and collaborators proposed that donkey semen extended with a milk-based extender containing glycerol used to breed mares is less sensitive to glycerol-toxicity than the same semen used to breed jennies, as the final concentration affecting the fertility of donkey semen in mares was ~5% and approximately 2% in jennies [28,29,30]. The EY extender used in the present study contained 1% of glycerol and 4% of formamide, approximately half of the toxic glycerol concentration proposed by Vidament et al. [28]. A report comparing ejaculated donkey semen extended in EY, SC and milk-based extender cooled for 24 h demonstrated that the EY extender resulted in superior sperm motility parameters [31].

A study demonstrated that epididymal semen has superior cooling ability than ejaculated stallion semen; this does not seem to be the case in donkeys [32]. While the present study did not compare donkey ejaculated sperm with donkey epididymal semen, the overall results of the semen parameters appear inferior to that expected with cooled ejaculated donkey semen [2]. However, it remains to be determined if cooled donkey epididymal semen extended in the EY used in the present study can result in satisfactory fertility when used to inseminate mares and jennies. It is also unknown if the same formula of the EY extender used herein deprived of glycerol and formamide would result in superior results than those obtained herein and by previous authors. Finally, donkey semen extended in EY-based extender seems to behave similarly to ruminant semen regarding glycerol exposure during cooling.

While the present design was not set to compare semen in the three different experiments, a subjective comparison across experiments seems to trend towards a reduction in TM and PM between the freshly harvested epididymal semen and the cooled-shipped semen. In stallions, it has been shown that epididymal semen can be stored at 5 °C for up to 96 h before being processed [19]. However, the apparent differences could have also been due to the different sample sizes, husbandry practices, levels of stress, weather, and other potential confounding factors not identified. The shipment of the entire scrotal content (Experiment 2) rather than the dissected epididymides (Experiment 3) seemed to yield superior sperm parameters; however, the comparison was not included in the statistical analyses. Several variables can be accounted for the different results. The temperature for both passive cooling containers was equivalent both on the day of arrival and 24 h later. The donkeys included in Experiment 2 appeared to be more mature than the donkeys used in Experiment 3 and were more likely to be kept in a more stable environment. While a cross-over design involving both passive cooling containers were not tested, the authors believe that the apparent different results cannot be accounted for by the type of container. It is also unclear whether this difference is associated with sample preparation. Upon arrival, both types of containers had a similar internal temperature; due to each container’s different features, it is reasonable to suggest that the cooling curve might have been different. In Experiment 2, the testes were placed in direct contact with the iced can, without the use of an isothermolizer cup. It is reasonable to assume that the presence of the testicles inside the shipper could have mitigated the reduction temperature, creating a less steep cooling curve. Further investigations are needed to evaluate the effect of different containers and cooling curves on the quality of the semen harvested from the epididymis.

The results of the present study with the donkey epididymal semen parameters were lower than those reported in the literature with raw or frozen-thawed donkey ejaculated semen [2]. Interestingly, there was an overall high percentage of PMI with HMMP. Across mammalian species, sperm stored at the tail of the epididymis is immotile due to plasma membrane-bound proteins that prevent premature motility and loss of viability [33,34]. In stallions, the EY extender used in the present study was shown to activate the motility of epididymal semen [8]; thus, the protocol of directly harvesting epididymal semen with this extender became popular in equine clinical practice and has been used in numerous investigations [6,32]. Semen centrifugation has been used as a means to potentially activate epididymal sperm motility in mice by removing plasma membrane-bound proteins [35]. In addition, centrifugation is a mandatory step for the cryopreservation of ejaculated semen of a jack or stallion to improve sperm interaction with cryoprotectants by removing the seminal plasma [2,9]. However, sperm harvested from the epididymis does not have seminal plasma; therefore, the need for centrifugation is questionable, as a very high sperm concentration is often yielded. Some authors do not recommend centrifugation before freezing epididymal semen in stallions [6,18]. One study found no differences in the post-thaw parameters for semen flushed and frozen with the EY extender used herein or semen flushed with a milk-based extender, cushion-centrifuged and then extended and frozen in the same EY extender [6]. Our results in the present experiment appear to concur with the latter study; in fact, it appears to suggest that centrifugation may not be beneficial for donkey epididymal semen cooling or freezing. However, since we did not centrifuge semen flushed with the EY extender, it is possible that some of the differences observed herein could have been simply the difference between extenders rather than centrifugation. In addition, since semen freezing extenders are more expensive than cooling extenders, the use of EY could be cost-prohibitive in practice; thus, this approach was not tested. In addition, the sperm loss associated with traditional centrifugation (600 g × 10 min, ~40%) or cushioned-centrifugation (1000 g × 20 min, ~10%) may be discouraging in using either approach as the last chance to cryopreserve semen from a valuable stallion [21].

## 5. Conclusions

In conclusion, freshly harvested, cooled-shipped, and cooled semen had satisfactory semen parameters. The post-thaw results revealed low motility parameters across any type of extender or methods of processing; unexpectedly, the HMMP and PMI did not reflect this trend, and the values remained high, suggesting that there was a lack of epididymal sperm activation with either centrifugation or extenders. While fertility was not tested in the present study, our in vitro results with cooled epididymal semen are encouraging and suggest that donkey epididymal semen may have satisfactory fertility. New studies need to address the fertility of donkey epididymal semen in mares and jennies.

## Figures and Tables

**Table 1:** Donkey epididymal semen parameters were assessed immediately after semen harvesting (pre-cooling) or after cooling for 24 h (post-cooling). Harvesting was performed within 1 h post-castration (*n* = 4 pairs). Each epididymal pair was submitted to retrograde flushing with a freezing egg yolk-based extender (EY) or a cooling extender based on sodium caseinate cholesterol-loaded cyclodextrin (SC). Thereafter, the semen extended in EY was cooled for 24 h, whereas the semen extended in SC was cooled for 24 h, or cushion-centrifuged, re-extended with SC, and then cooled for 24 h (C-SC). Data are expressed as mean ± SEM.

	EY		SC		C-SC	
	Pre-Cooling	Post-Cooling	Pre-Cooling	Post-Cooling	Pre-Cooling	Post-Cooling
**TM**	88.0 ± 0.6	71.5 ± 4.4	47.0 ± 15.6	51.6 ± 8.6	57.2 ± 13.3	54.4 ± 8.8
**PM**	81.8 ± 0.9	63.8 ± 4.1	37.4 ± 14.7	39.3 ± 8.5	44.7 ± 13.6	40.1 ± 10.4
**VSL**	66.8 ± 3.3	55.1 ± 7.1	55.3 ± 8.6	43.6 ± 2.4	52.7 ± 6.0	38.8 ± 1.4
**VCL**	153.5 ± 4.8	123.5 ± 9.3	135.6 ± 13.4	118.5 ± 11.8	131.9 ± 11.8	112.5 ± 7.2
**VAP**	82.0 ± 2.7	65.2 ± 6.9	67.9 ± 7.9	56.4 ± 3.0	65.5 ± 6.6	50.8 ± 0.9
**PMI**	91.1 ± 0.8	84.0 ± 4.5	86.9 ± 3.6	80.2 ± 8.7	86.3 ± 0.7	86.2 ± 0.7
**HMMP**	94.0 ± 1.8	93.5 ± 1.9	89.0 ± 2.0	93.7 ± 1.4	91.5 ± 1.5	91.9 ± 0.9

**TM**, total motility (%); **PM**, progressive motility (%); **VAP**, average path velocity (µm/s); **VCL**, curvilinear velocity (µm/s); **VSL**, straight-line velocity (µm/s); **PMI**, plasma membrane integrity (%); **HMMP**, sperm with intact plasma membrane and high mitochondrial membrane potential (%).

**Table 2:** Pre-freeze and post-thaw parameters of the epididymal semen obtained from freshly castrated donkeys (*n* = 4 pairs). Each epididymal pair was submitted to retrograde flushing with a semen freezing egg yolk-based extender (EY), or a cooling extender based on sodium caseinate cholesterol-loaded cyclodextrin (SC). Thereafter, the semen extended in EY was frozen immediately after recovery (**Non-Centrif-0 h**) or cooled for 24 h and then frozen (**Non-Centrif-24 h**). The semen extended in **SC** was cushion-centrifuged, resuspended in EY and then frozen (**Centrif-0 h**); or cooled for 24 h before being frozen (**Centrif-24 h**); or cushion-centrifuged, re-extended with SC, and then cooled for 24 h, re-centrifuged and extended **in EY** and then frozen (**C-Centrif-24 h**). Samples were assessed **pre-freezing** and **post-freezing**. Data are expressed as mean ± SEM.

		Non-Centrif		Centrif		C-Centrif
		0 h	24 h	0 h	24 h	24 h
Pre-Freezing	**TM**	88.0 ± 0.6 ^A^	71.5 ± 4.4 ^A^	86.4 ± 4.4 ^Aa^	72.7 ± 4.6 ^Ab^	75.3 ± 3.7 ^A^
	**PM**	81.8 ± 0.9 ^A^	63.8 ± 4.1 ^A^	80.2 ± 7.4 ^Aa^	64.0 ± 5.2 ^Ab^	64.4 ± 6.8 ^A^
	**VSL**	66.8 ± 3.3 ^Aa^	55.1 ± 7.1	67.6 ± 10.1 ^aA^	44.8 ± 0.8 ^b^	46.6 ± 2.2 ^b^
	**VCL**	153.5 ± 4.8 ^aA^	123.5 ± 9.3	146.8 ± 16.1 ^aA^	117.1 ± 1.2 ^b^	121.8 ± 5.8 ^b^
	**VAP**	82.0 ± 2.7 ^aA^	65.2 ± 6.9	82.2 ± 8.8 ^aA^	57.6 ± 1.0 ^b^	58.6 ± 3.3 ^b^
	**PMI**	91.1 ± 0.8	84.0 ± 4.5 ^A^	83.9 ± 6.0 ^A^	88.2 ± 0.9	86.6 ± 0.9
	**HMMP**	93.8 ± 1.8	93.5 ± 1.9	94.5 ± 0.8 ^A^	93.2 ± 3.8	92.9 ± 3.9
Post-Freezing	**TM**	35.2 ± 1.7 ^B^	31.9 ± 7.7 ^B^	22.3 ± 5.3 ^B^	39.8 ± 8.2 ^B^	36.2 ± 2.2 ^B^
	**PM**	26.9 ± 1.3 ^B^	24.6 ± 6.8 ^B^	15.4 ± 3.4 ^B^	31.3 ± 5.9 ^B^	27.0 ± 1.8 ^B^
	**VSL**	40.2 ± 7.3 ^B^	39.1 ± 0.4	40.3 ± 0.2 ^B^	36.9 ± 2.5	37.1 ± 0.7
	**VCL**	111.1 ± 16.6 ^B^	99.8 ± 4.9	118.4 ± 1.2 ^B^	93.82 ± 2.2	97.3 ± 9.1
	**VAP**	52.0 ± 7.0 ^aB^	48.0 ± 0.6	52.5 ± 4.0 ^aB^	45.2 ± 2.0 ^b^	45.3 ± 1.3 ^b^
	**PMI**	70.0 ± 8.6	56.7 ± 10.6^B^	47.2 ± 7.3 ^B^	68.8 ± 2.2	67.9 ± 3.2
	**HMMP**	90.3 ± 0.9	91.0 ± 1.9	78.9 ± 3.7 ^B^	89.5 ± 4.2	87.6 ± 1.9

**TM**, total motility (%); **PM**, progressive motility (%); **VAP**, average path velocity (µm/s); **VCL**, curvilinear velocity (µm/s); **VSL**, straight-line velocity (µm/s); **PMI**, plasma membrane integrity (%); **HMMP**, sperm with intact plasma membrane and high mitochondrial membrane potential (%). Different superscripts denote differences within (^AB^) or between (^ab^) columns. (*p* < 0.05).

**Table 3:** Sperm velocity and viability parameters of donkey epididymal semen cooling harvested from cooled shipped epididymides. The scrotal content was shipped in a passive cooling semen container. The epididymides were processed upon arrival (*n* = 8 pairs, **cooled-shipped 24 h**) or the following day (*n* = 6 pairs, **cooled-shipped 48 h**). All epididymides were kept in the passive cooling device until processing. For each of the time points, one epididymis of each pair was submitted to retrograde flushing with a freezing egg yolk-based extender (**EY**), while the contralateral epididymis was flushed with a cooling extender based on sodium caseinate cholesterol-loaded cyclodextrin (**SC**). Thereafter, the semen extended in EY was cooled for 24 h; whereas the semen extended in **SC** was cooled for 24 h, or cushion-centrifuged, re-extended with SC, and then cooled for 24 h (**C-SC**). Semen was assessed immediately after harvesting (**pre-cooling**) and after cooling for 24 h (**post-cooling**). Data are expressed as the mean ± SEM.

		Cooled-Shipped 24 h			Cooled-Shipped 48 h		
		EY	SC	C-SC	EY	SC	C-SC
Pre-Cooling	**TM**	67.7 ± 7.5	55.6 ± 7.6	65.6 ± 7.4	67.6 ± 5.7	58.3 ± 12.7	73.3 ± 7.1
	**PM**	58.0 ± 8.2	44.2 ± 7.0	59.5 ± 7.9	58.8 ± 6.6	52.1 ± 11.9	66.0 ± 6.7
	**VSL**	58.8 ± 4.3	49.1 ± 3.9	66.9 ± 3.8	55.0 ± 1.4	59.7 ± 5.0	68.0 ± 4.0
	**VCL**	134.5 ± 8.8	121.0 ± 4.4	149.2 ± 5.0	143.2 ± 3.2	146.5 ± 7.2	148.8 ± 2.6
	**VAP**	71.7 ± 4.6	62.0 ± 3.3	80.7 ± 4.0	64.7 ± 5.5	69.2 ± 3.2	68.8 ± 2.8
	**PMI**	78.5 ± 8.9	87.0 ± 2.2	83.8 ± 2.4	82.1 ± 10.0	85.7 ± 2.7	86.8 ± 1.1
	**HMMP**	94.2 ± 1.6	88.9 ± 2.4	91.2 ± 1.5	75.2 ± 18.8	89.0 ± 3.4	88.2 ± 2.5
Post-Cooling	**TM**	64.0 ± 6.2	40.0 ± 8.2	44.0 ± 7.6	71.3 ± 1.3	60.4 ± 9.7	60.8 ± 8.6
	**PM**	55.8 ± 8.3	33.8 ± 7.7	37.1 ± 7.6	64.1 ± 1.5	53.8 ± 9.2	56.1 ± 7.2
	**VSL**	50.8 ± 5.3	56.6 ± 3.3	57.4 ± 3.3	55.1 ± 7.7	61.9 ± 2.2	61.9 ± 2.4
	**VCL**	138.1 ± 13.5	136.7 ± 4.0	135 ± 1.9	133.4 ± 19.0	145.1 ± 3.1	139.1 ± 5.0
	**VAP**	70.6 ± 2.1	75.4 ± 4.8	83.5 ± 4.1	71.4 ± 7.6	76.0 ± 1.4	73.1 ± 2.3
	**PMI**	84.8 ± 6.1	82.9 ± 4.1	78.6 ± 4.1	90.2 ± 1.3	83.4 ± 2.5	75.8 ± 3.4
	**HMMP**	88.6 ± 2.9	86.9 ± 2.1	78.9 ± 7.6	91.5 ± 4.7	88.3 ± 1.7	84.3 ± 4.2

**TM**, total motility (%); **PM**, progressive motility (%); **VAP**, average path velocity (µm/s); **VCL**, curvilinear velocity (µm/s); **VSL**, straight-line velocity (µm/s); **PMI**, plasma membrane integrity (%); **HMMP**, sperm with intact plasma membrane and high mitochondrial membrane potential (%).

**Table 4:** Pre- and post-freezing parameters of the epididymal semen obtained from donkey cooled-shipped epididymides attached to the testes. All epididymides were kept in the passive cooling device until processing. The epididymides were processed upon arrival (*n* = 8 pairs) (**cooled-shipped 24 h**) or the following day (*n* = 6 pairs) (**cooled-shipped 48 h**). One epididymis of each pair was submitted to retrograde flushing with a semen freezing egg yolk-based extender (EY), while the contralateral was flushed with a cooling extender based on sodium caseinate cholesterol-loaded cyclodextrin (SC). Thereafter, the semen extended in EY was frozen immediately after recovery (**Non-Centrif-0 h**) or cooled for 24 h and then frozen (**Non-Centrif-24 h**). The semen extended in SC was cushion-centrifuged, resuspended in EY and then frozen (**Centrif-0 h**); or cooled for 24 h before being frozen (**Centrif-24 h**); or cushion-centrifuged, re-extended with SC, and then cooled for 24 h, re-centrifuged and extended in EY and then frozen (**C-Centrif-24 h**). Data are expressed as mean ± SEM.

		Cooled-Shipped 24 h					Cooled-Shipped 48 h				
		Non-Centrif		Centrif		C-Centrif	Non-Centrif		Centrif		C-Centrif
		0 h	24 h	0 h	24 h	24 h	0 h	24 h	0 h	24 h	24 h
Pre-Freezing	**TM**	67.7 ± 7.5 ^A^	64.0 ± 6.2 ^A^	74.6 ± 5.3 ^A^	68.3 ± 6.4 ^A^	59.5 ± 6.8 ^A^	67.6 ± 5.7 ^A^	71.3 ± 1.3 ^A^	72.7 ± 8.8 ^A^	64.3 ± 7.6 ^A^	64.4 ± 9.0 ^A^
	**PM**	58.0 ± 8.2 ^A^	55.8 ± 8.4 ^A^	68.6 ± 5.5 ^A^	61.2 ± 7.9 ^A^	50.0 ± 8.9 ^A^	58.8 ± 6.6	64.1 ± 1.5	67.0 ± 8.8	57.8 ± 7.8	57.9 ± 8.8
	**VSL**	58.8 ± 4.3	50.9 ± 5.3	60.0 ± 2.8	54.5 ± 5.7	54.5 ± 4.5	55.0 ± 1.4	55.1 ± 7.7	56.6 ± 2.8	53.2 ± 5.8	53.7 ± 5.3
	**VCL**	134.5 ± 8.8	138.1±13.5	134.1 ± 6.9	131.2 ± 6.6	123.8 ± 5.9	143.2 ± 3.2	133.4 ± 19.1	132.6 ± 4.7	126.6 ± 7.3	121.7 ± 9.3
	**VAP**	71.7 ± 4.6	64.6 ± 5.5	71.6 ± 2.9	67.9 ± 5.7	65.8 ± 4.4	70.6 ± 2.1	71.4 ± 7.6	70.6 ± 3.8	64.9 ± 6.3	64.5 ± 6.1
	**PMI**	78.5 ± 8.9	84.8 ± 6.1	87.6 ± 3.8	82.6 ± 7.9	85.0 ± 1.7	82.1 ± 10.0	90.2 ± 1.3	90.0 ± 4.2	85.8 ± 1.1	74.3 ± 6.6
	**HMMP**	94.2 ± 1.6	88.6 ± 2.9	87.8 ± 3.9	72.5 ± 8.2	86.5 ± 2.7	75.2 ± 18.8	91.5 ± 4.7	91.8 ± 1.8	88.2 ± 4.6	82.7 ± 5.2
Post-Freezing	**TM**	22.8 ± 4.1 ^B^	19.6 ± 4.9 ^B^	30.8 ± 4.3 ^B^	22.1 ± 2.9 ^B^	25.2 ± 4.0 ^B^	20.4 ± 3.0 ^B^	25.1 ± 4.2 ^B^	25.6 ± 3.4 ^B^	26.0 ± 0.1 ^B^	20.2 ± 3.5 ^B^
	**PM**	15.2 ± 4.1 ^B^	14.5 ± 4.7 ^B^	21.1 ± 3.5 ^B^	12.6 ± 2.3 ^B^	13.9 ± 3.0 ^B^	11.5 ± 3.1 ^B^	15.1 ± 3.4 ^B^	15.4 ± 3.4 ^B^	13.4 ± 1.1 ^B^	10.5 ± 2.3 ^B^
	**VSL**	40.6 ± 2.8	42.5 ± 4.2	46.1 ± 1.5	36.1 ± 3.1	40.3± 2.6	45.7 ± 1.4	41.0 ± 4.1	40.6 ± 2.4	38.1 ± 0.3	38.9 ± 1.5
	**VCL**	100.8 ± 6.5	100.6 ± 12.8	118.0 ± 3.2	99.7 ± 4.8	107.9 ± 3.2	127.4 ± 3.1	116.6 ± 6.5	112.5 ± 5.3	103.5 ± 1.4	109.5 ± 3.5
	**VAP**	50.2 ± 2.8	51.3 ± 5.0	56.3 ± 1.3	45.7 ± 3.3	51.1 ± 2.5	59.2 ± 1.5	51.8 ± 4.4	50.7 ± 3.2	46.6 ± 0.7	49.4 ± 2.1
	**PMI**	46.5 ± 5.4	41.0 ± 9.4	63.6 ± 4.3	59.2 ± 3.3	57.7 ± 3.2	51.7 ± 4.3	46.6 ± 9.7	46.3 ± 5.4	60.0 ± 4.7	58.7 ± 7.4
	**HMMP**	87.5 ± 3.2	87.3 ± 3.0	91.5 ± 1.6	94.0 ± 1.1	91.7 ± 1.7	86.6 ± 4.6	90.3 ± 2.5	90.0 ± 5.0	91.6 ± 6.5	72.5 ± 21.5

**TM**, total motility (%); **PM**, progressive motility (%); **VAP**, average path velocity (µm/s); **VCL**, curvilinear velocity (µm/s); **VSL**, straight-line velocity (µm/s); **PMI**, plasma membrane integrity (%); **HMMP**, sperm with intact plasma membrane and high mitochondrial membrane potential (%). Different superscripts denote differences within (^AB^) columns. (*p* < 0.05).

**Table 5:** Motility and viability parameters of the donkey epididymal semen harvested from cooled-shipped epididymides dissected away from the testes. The epididymides were processed upon arrival (*n* = 20 pairs, **cooled-shipped 24 h**) or the following day post-arrival (*n* = 16 pairs, **cooled-shipped 48 h**). All epididymides were kept in the passive cooling device until processing. For each of the time points, each epididymal pair was submitted to retrograde flushing with a freezing egg yolk-based extender (**EY**), or a cooling extender based on sodium caseinate cholesterol-loaded cyclodextrin (**SC**). Thereafter, the semen extended in EY was cooled for 24 h; whereas the semen extended in SC was cooled for 24 h, or cushion-centrifuged, re-extended with SC, and then cooled for 24 h (**C-SC**). Semen was assessed immediately after harvesting (**pre-cooling**) and after cooling for 24 h (**post-cooling**). Data are expressed as the mean ± SEM.

		Cooled-Shipped 24 h			Cooled-Shipped 48 h		
		EY	SC	C-SC	EY	SC	C-SC
Pre-Cooling	**TM**	43.0 ± 3.9 ^a^	22.3 ± 4.5 ^b^	25.9 ± 5.2 ^b^	43.3 ± 4.7 ^a^	21.7 ± 3.1 ^b^	18.7 ± 2.4 ^b^
	**PM**	31.7 ± 3.6 ^aA^	15.3 ± 4.2 ^b^	16.6 ± 5.0 ^b^	32.8 ± 4.7 ^aA^	12.9 ± 2.3 ^b^	10.2 ± 1.8 ^b^
	**VSL**	44.0 ± 1.9	35.0 ± 4.0	38.1 ± 3.1	45.0 ± 2.1	38.3 ± 2.7	45.7 ± 3.6
	**VCL**	107.5 ± 3.8 ^a^	79.3 ± 9.1 ^b^	98.4 ± 5.5	114.1 ± 5.1	97.8 ± 6.2	105.0 ± 6.8
	**VAP**	53.6 ± 2.1	42.6 ± 4.6	46.8 ± 3.4	56.8 ± 2.7	48.6 ± 3.1	54.9 ± 4.0
	**PMI**	76.0 ± 4.2	72.5 ± 4.0 ^a^	74.0 ± 2.5	57.5 ± 5.0 ^A^	54.9 ± 6.2 ^b^	44.8 ± 4.5
	**HMMP**	80.3 ± 5.4	82.9 ± 4.7	77.3 ± 6.8	84.3 ± 4.5	83.6 ± 3.6	83.0 ± 3.9
Post-Cooling	**TM**	56.8 ± 5.9	21.9 ± 3.8	25.6 ± 4.5	53.6 ± 5.2	24.3 ± 2.8	23.0 ± 2.9
	**PM**	48.1 ± 5.7 ^B^	13.8 ± 3.1	15.1 ± 3.7	44.1 ± 4.9 ^B^	15.4 ± 2.3	11.7 ± 2.0
	**VSL**	44.9 ± 2.3	36.2 ± 2.3	35.8 ± 2.2	42.6 ± 1.6	36.1 ± 2.7	43.5 ± 3.8
	**VCL**	111.0 ± 5.0	83.4 ± 6.6	94.2 ± 4.0	110.2 ± 5.1	87.9 ± 6.1	100.8 ± 3.0
	**VAP**	56.9 ± 2.8	44.5 ± 2.7	44.8 ± 2.6	54.5 ± 2.4	45.6 ± 3.1	52.8 ± 3.5
	**PMI**	78.9 ± 4.2	65.8 ± 6.1	72.0 ± 5.8	77.6 ± 2.9 ^B^	61.9 ± 4.3	62.5 ± 2.4
	**HMMP**	88.9 ± 2.5	75.8 ± 6.7	83.8 ± 5.2	87.6 ± 2.1	81.8 ± 2.6	78.2 ± 5.4

**TM**, total motility (%); **PM**, progressive motility (%); **VAP**, average path velocity (µm/s); **VCL**, curvilinear velocity (µm/s); **VSL**, straight-line velocity (µm/s); **PMI**, plasma membrane integrity (%); **HMMP**, sperm with intact plasma membrane and high mitochondrial membrane potential (%). Different superscripts denote differences within (^AB^) or between (^ab^) columns. (*p* < 0.05).

**Table 6:** Pre- and post-freezing parameters of the epididymal semen obtained from donkey cooled-shipped epididymides (**cooled-shipped 24 h**) dissected from the testes. All epididymides were kept in the passive cooling device until processing. The epididymides were processed upon arrival (*n* = 20 pairs, **cooled-shipped 24 h**) or the following day post-arrival (*n* = 16 pairs, **cooled-shipped 48 h**). Each epididymal pair was submitted to retrograde flushing with a semen freezing egg yolk-based extender (EY), or a cooling extender based on sodium caseinate cholesterol-loaded cyclodextrin (SC). Thereafter, the semen extended in EY was frozen immediately after recovery (**Non-Centrif-0 h**) or cooled for 24 h and then frozen (**Non-Centrif-24 h**). The semen extended in SC was cushion-centrifuged, resuspended in EY and then frozen (**Centrif-0 h**); or cooled for 24 h before being frozen (**Centrif-24 h**); or cushion-centrifuged, re-extended with SC, and then cooled for 24 h, re-centrifuged and extended in EY and then frozen (**C-Centrif-24 h**). Data are expressed as the mean ± SEM.

		Cooled-Shipped 24 h					Cooled-Shipped 48 h				
		Non-Centrif		Centrif		C-Centrif	Non-Centrif		Centrif		C-Centrif
		0 h	24 h	0 h	24 h	24 h	0 h	24 h	0 h	24 h	24 h
Pre-Freezing	**TM**	43.0 ± 3.9 ^A^	56.8 ± 5.9 ^A^	43.9 ± 4.1 ^A^	43.9 ± 4.2 ^A^	50.8 ± 4.8 ^A^	43.3 ± 4.7 ^A^	53.6 ± 5.2 ^Aa^	49.0 ± 4.1 ^A^	36.0 ± 4.8 ^Ab^	39.7 ± 3.9 ^A^
	**PM**	31.7 ± 3.6 ^aA^	48.0 ± 5.7 ^Ab^	33.2 ± 4.3 ^A^	30.7 ± 4.0 ^Aa^	37.2 ± 4.7 ^A^	32.8 ± 4.7 ^A^	44.1 ± 4.9 ^Aa^	39.6 ± 4.3 ^A^	27.1 ± 4.1 ^Ab^	27.0 ± 3.8 ^A^
	**VSL**	44.0 ± 1.9	44.9 ± 2.3 ^A^	44.8 ± 2.5 ^Aa^	37.0 ± 1.5 ^Ab^	40.3 ± 1.9	45.2 ± 2.1 ^A^	42.6 ± 1.6 ^A^	47.5 ± 2.1 ^A^	35.8 ± 2.3	36.3 ± 1.8
	**VCL**	107.5 ± 3.8 ^A^	111.0 ± 5.0 ^A^	108.8 ± 4.7 ^Aa^	101.6 ± 3.7 ^Ab^	105.7 ± 3.3	114.1 ± 5.1	110.2 ± 5.1 ^A^	114.7 ± 4.5 ^a^	93.0 ± 7.1 ^Bb^	95.2 ± 4.0 ^Bb^
	**VAP**	53.6 ± 2.1	56.9 ± 2.7 ^A^	54.6 ± 2.7 ^A^	48.3 ± 2.0 ^B^	51.2 ± 2.2	56.8 ± 2.7 ^A^	54.5 ± 2.4 ^A^	58.9 ± 2.5 ^Aa^	46.4 ± 2.9 ^b^	46.7 ± 2.1 ^b^
	**PMI**	76.0 ± 4.2	76.0 ± 4.2 ^A^	76.8 ± 2.4 ^A^	68.7 ± 7.0 ^A^	72.8 ± 9.3	57.5 ± 5.0 ^a^	77.6 ± 2.9 ^b^	61.9 ± 3.8 ^A^	69.1 ± 4.6 ^A^	73.6 ± 2.7 ^A^
	**HMMP**	80.3 ± 5.4 ^a^	80.3 ± 5.4 ^a^	52.8 ± 10.1 ^b^	76.4 ± 7.5 ^a^	91.0 ± 2.0 ^a^	84.3 ± 4.5	87.6 ± 2.1	76.2 ± 5.7	83.0 ± 5.4	90.3 ± 1.6
Post-Freezing	**TM**	17.6 ± 1.3 ^B^	22.5 ± 2.3 ^B^	20.4 ± 2.0 ^B^	15.7 ± 2.1 ^B^	20.3 ± 2.0 ^B^	13.3 ± 1.2 ^B^	19.8 ± 2.1 ^B^	20.0 ± 2.2 ^B^	14.2 ± 1.6 ^B^	17.5 ± 1.7 ^B^
	**PM**	10.6 ± 1.3 ^B^	15.1 ± 2.2 ^B^	11.0 ± 1.5 ^B^	8.0 ± 1.8 ^B^	10.5 ± 1.6 ^B^	6.9 ± 1.0 ^B^	12.9 ± 2.1 ^B^	11.9 ± 1.5 ^B^	6.7 ± 1.2 ^B^	8.5 ± 1.1 ^B^
	**VSL**	36.3 ± 1.7 ^B^	33.6 ± 1.7 ^B^	33.9 ± 1.6 ^B^	28.6 ± 2.0 ^B^	34.0 ± 1.3	33.9 ± 1.4 ^B^	34.0 ± 1.3 ^B^	34.7 ± 0.9 ^B^	30.6 ± 1.5	32.4 ± 0.9
	**VCL**	88.9 ± 3.7	84.4 ± 4.6 ^B^	87.7 ± 4.4 ^B^	72.0 ± 7.2 ^B^	92.9 ± 2.1	94.5 ± 3.7	81.7 ± 4.6 ^B^	95.4 ± 3.1	80.4 ± 4.6	90.9 ± 3.7
	**VAP**	44.1 ± 1.9 ^B^	41.1 ± 1.8 ^B^	41.4 ± 1.8 ^B^	35.5 ± 2.7 ^B^	42.2 ± 1.3	42.4 ± 1.5 ^B^	40.7 ± 1.6 ^B^	44.0 ± 1.2 ^B^	38.8 ± 1.5	42.5 ± 1.2
	**PMI**	44.8 ± 3.1 ^B^	41.0 ± 2.1 ^B^	53.6 ± 3.1 ^B^	41.4 ± 4.8 ^B^	53.3 ± 2.9	44.7 ± 2.7	49.3 ± 3.8	50.3 ± 2.3 ^B^	47.6 ± 3.7 ^B^	53.4 ± 2.5 ^B^
	**HMMP**	83.0 ± 2.0	84.4 ± 2.9	86.4 ± 1.9	79.1 ± 4.8	83.7 ± 4.7	82.6 ± 2.7	90.7 ± 1.5	86.8 ± 2.2	80.3 ± 6.2	87.0 ± 2.2

**TM**, total motility (%); **PM**, progressive motility (%); **VAP**, average path velocity (µm/s); **VCL**, curvilinear velocity (µm/s); **VSL**, straight-line velocity (µm/s); **PMI**, plasma membrane integrity (%); **HMMP**, sperm with intact plasma membrane and high mitochondrial membrane potential (%). Different superscripts denote differences within (^AB^) or between (^ab^) columns. (*p* < 0.05).

## Supplementary Materials

The following are available online at https://www.mdpi.com/2076-2615/10/12/2209/s1, Table S1: Donkey ages, tail of epididymis weight, and total sperm recovery harvested freshly after castration; Table S2: Donkey age, tail of epididymis weight, total sperm recovery harvested and temperature of the container at the opening. Scrotal content was shipped in passive cooling semen containers. Donkeys 1–8 were processed after arrival (Cooled-shipped 24 h), whereas donkeys 9–14 were processed 24 h later (Cooled-shipped 48 h); Table S3: Donkey age, tail of epididymis weight, total sperm recovery harvested, and temperature of the container at opening. Epididymides were shipped in passive cooling semen containers. Donkeys 1–20 were processed after arrival (Cooled-shipped 24 h), whereas donkeys 21–36 were processed 24 h later (Cooled-shipped 48 h).

## References

[1] Canisso I.F., Mcdonnell S.M. (2010). Donkey breeding behavior with an emphasis on the Pêga breed. Vet. Care Donkeys Int. Vet. Inf. Serv..

[2] Canisso I.F., Panzani D., Miró J., Ellerbrock R.E. (2019). Key Aspects of Donkey and Mule Reproduction. Vet. Clin. N. Am. Equine Pract..

[3] Canisso I.F., Davies Morel M.C.G., McDonnell S. (2009). Strategies for the management of donkey jacks in intensive breeding systems. Equine Vet. Educ..

[4] Canisso I.F., Carvalho G.R., Morel M.D., Ker P.G., Rodrigues A.L., Silva E.C., Coutinho da Silva M.A. (2011). Seminal parameters and field fertility of cryopreserved donkey jack semen after insemination of horse mares. Equine Vet. J..

[5] Rota A., Panzani D., Sabatini C., Camillo F. (2012). Donkey jack (*Equus asinus*) semen cryopreservation: Studies of seminal parameters, post-breeding inflammatory response, and fertility in donkey jennies. Theriogenology.

[6] Ellerbrock R.E., Prell M.J., Stewart J.L., Bojko M.S., Lima F.S., Canisso I.F. (2017). Comparison of Centrifugation and Noncentrifugation Methods to Cryopreserve Stallion Epididymal Semen. J. Equine Vet. Sci..

[7] Braun J., Sakai M., Hochi S., Oguri N. (1994). Preservation of ejaculated and epididymal stallion spermatozoa. Theriogenology.

[8] Papa F.O., Melo C.M., Fioratti E.G., Dell’Aqua J.A., Zahn F.S., Alvarenga M.A. (2008). Freezing of stallion epididymal sperm. Anim. Reprod. Sci..

[9] Amann R.P., Pickett B.W. (1987). Principles of cryopreservation and a review of cryopreservation of stallion spermatozoa. J. Equine Vet. Sci..

[10] Bruemmer J.E. (2006). Collection and Freezing of Epididymal Stallion Sperm. Vet. Clin. N. Am. Equine Pract..

[11] Camillo F., Rota A., Biagini L., Tesi M., Fanelli D., Panzani D. (2018). The Current Situation and Trend of Donkey Industry in Europe. J. Equine Vet. Sci..

[12] Adams G.P., Ratto M.H., Collins C.W., Bergfelt D.R. (2009). Artificial insemination in South American camelids and wild equids. Theriogenology.

[13] Beek J., Maes D., Nauwynck H., Piepers S., van Soom A. (2015). A critical assessment of the effect of serine protease inhibitors on porcine fertilization and quality parameters of porcine spermatozoa in vitro. Reprod. Biol..

[14] Herold F.C., Aurich J.E., Gerber D. (2004). Epididymal sperm from the African buffalo (*Syncerus caffer*) can be frozen successfully with AndroMed^®^ and with Triladyl^TM^ but the addition of bovine seminal plasma is detrimental. Theriogenology.

[15] Mamani-Mango G., Gonzales M.M., Hidalgo M.R., Mallma J.M., Béjar J.R., Palma V.R., Mellisho Salas E. (2019). Effect of Extender and Freezing Rate on Quality Parameters and in Vitro Fertilization Capacity of Alpaca Spermatozoa Recovered from *Cauda Epididymis*. Biopreserv. Biobank.

[16] Vilela C.G., Marquez J.M., Graham J.K., Barfield J.P. (2017). Cryopreservation of bison epididymal sperm: A strategy for improving post-thaw quality when collecting sperm in field conditions. Theriogenology.

[17] Miró J., Morató R., Vilagran I., Taberner E., Bonet S., Yeste M. (2020). Preservation of Epididymal Stallion Sperm in Liquid and Frozen States: Effects of Seminal Plasma on Sperm Function and Fertility. J. Equine Vet. Sci..

[18] Stawicki R.J. (2015). How to Process Stallion Epididymal Sperm in Your Practice. Am. Assoc. Equine Pract..

[19] Stawicki R.J., McDonnell S.M., Giguère S., Turner R.M. (2016). Pregnancy outcomes using stallion epididymal sperm stored at 5 °C for 24 or 48 h before harvest. Theriogenology.

[20] Waite J.A., Love C.C., Brinsko S.P., Teague S.R., Salazar J.L., Mancill S.S., Varner D.D. (2008). Factors impacting equine sperm recovery rate and quality following cushioned centrifugation. Theriogenology.

[21] Bliss S.B., Voge J.L., Hayden S.S., Teague S.R., Brinsko S.P., Love C.C., Varner D.D. (2012). The impact of cushioned centrifugation protocols on semen quality of stallions. Theriogenology.

[22] Auer J., Stick J. (2012). Equine Surgery.

[23] Peña F.J., Ball B.A., Squires E.L. (2018). A New Method for Evaluating Stallion Sperm Viability and Mitochondrial Membrane Potential in Fixed Semen Samples. Cytom. Part B Clin. Cytom..

[24] Oliveira R.R., Rates D.M., Pugliesi G., Ker P.G., Arruda R.P., Moraes E.A., Carvalho G.R. (2014). Use of cholesterol-loaded cyclodextrin in donkey semen cryopreservation improves sperm viability but results in low fertility in mares. Reprod. Domest. Anim..

[25] Novello G., Podico G., Segabinazzi L.G.T.M., Lima F.S., Canisso I.F. (2020). Stallion Semen Cooling Using Native Phosphocaseinate-based Extender and Sodium Caseinate Cholesterol-loaded Cyclodextrin-based Extender. J. Equine Vet. Sci..

[26] Hartwig F.P., Lisboa F.P., Hartwig F.P., Monteiro G.A., Maziero R.R.D., Freitas-Dell’Aqua C.P., Alvarenga M.A., Papa F.O., Dell’Aqua J.A. (2014). Use of cholesterol-loaded cyclodextrin: An alternative for bad cooler stallions. Theriogenology.

[27] Pamornsakda T., Pojprasath T., Suwimonteerabutr J., Tharasanit T. (2011). Effects of cholesterol-loaded cyclodextrins on the quality of frozen-thawed equine epididymal sperm. Cryobiology.

[28] Vidament M., Vincent P., Yvon J.M., Bruneau B., Martin F.X. (2005). Glycerol in semen extender is a limiting factor in the fertility in asinine and equine species. Anim. Reprod. Sci..

[29] Vidament M., Vincent P., Martin F.X., Magistrini M., Blesbois E. (2009). Differences in ability of jennies and mares to conceive with cooled and frozen semen containing glycerol or not. Anim. Reprod. Sci..

[30] Saragusty J., Lemma A., Hildebrandt T.B., Göritz F. (2017). Follicular size predicts success in artificial insemination with frozen-Thawed sperm in donkeys. PLoS ONE.

[31] Rodrigues L.T., Canuto L.E.F., Oliveira S.N., Andrade L.R.P., Alvarenga M.A., Dell’Aqua J.A., Canisso I.F., Papa F.O. (2018). Skim Milk, Casein Based Extender or Botucrio in Cooled Semen Asinus (*Equus asinus*). J. Equine Vet. Sci..

[32] Monteiro G.A., Guasti P.N., Hartwig F.P., Dellaqua J.A., Alvarenga M.A., Papa F.O. (2013). Cooling of ejaculated and epididymal stallion sperm. Arq. Bras. Med. Vet. Zootec..

[33] Colenbrander B., Brouwers J.F.H.M., Neild D.M., Stout T.A.E., da Silva P., Gadella B.M. (2002). Capacitation dependent lipid rearrangements in the plasma membrane of equine sperm. Theriogenology.

[34] Combes G., Varner D., Schroeder F., Burghardt R., Blanchard T. (2000). Effect of cholesterol on the motility e plasma membrane integrity of frozen equine spermatozoa after thawing. J. Reprod. Fertil. Suppl..

[35] Carneiro J.A.M., Monteiro G.A., Martin I., Maziero R.R.D., Sancler-Silva Y.F.R., de Freitas-Dell’Aqua C.P., Guasti P.N., Bandeira R., Hartwig F.P., Ignácio F.S. (2017). Heterologous Oviductal Cells Binding Capacity of Cryopreserved Equine Ejaculated and Epididymal Spermatozoa. J. Equine Vet. Sci..

