# Supplementary material for: Donkey Epididymal Transport for Semen Cooling and Freezing
Source: Animals (Basel). 2020 Nov 25;10(12):2209. doi: 10.3390/ani10122209 (PMC7759917; doi:10.3390/ani10122209)
Supplement: Supplementary file 1 [file animals-10-02209-s001.pdf]

Table S1. Donkey ages, tail of epididymis weight, and total sperm recovery harvested freshly after castration.

Donkey	Age (yrs.)	Weight (g)	Extender	Total sperm (x10^9)
1	9	23.3	SC	18.1
		25.1	EY	14.9
2	2	14.9	SC	6.4
		15.4	EY	7
3	2	n/a	SC	n/a
		n/a	EY	n/a
4	10	11.0	SC	5.1
		10.1	EY	6.0
**Mean ± SEM**	5.7	16.7 ± 2.5		9.6 ± 2.2
**Range**	2-10	10.1-25.1		5.1-18.1

Table S2. Donkey age, tail of epididymis weight, total sperm recovery harvested and temperature of the container at opening. Scrotal content was shipped in passive cooling semen containers. Donkeys 1-8 were processed after arrival (**Cooled-shipped 24h**), whereas donkeys 9-14 were processed 24 h later (**Cooled-shipped 48 h**).

	Donkey	Age (yrs.)	Weight (g)	Extender	Total sperm ($x10^{9}$)	Temperature (°C)
**Cooled-shipped 24h**	1	6	28.2	SC	1.1	5.0
			31.0	EY	0.4	
	2	6	52.6	SC	5.1	5.0
			56.7	EY	10	
	3	4	22.0	SC	7.3	6.9
			22.3	EY	5.4	
	4	4	23.9	SC	9.6	6.9
			21.0	EY	7.9	
	5	6	30.2	SC	6.5	9.7
			30.8	EY	6.4	
	6	5	30.4	SC	9.0	9.7
			30.9	EY	4.6	
	7	8	12.7	SC	4.9	9.5
			10.8	EY	5.0	
	8	12	15.4	SC	12.8	8.4
			13.7	EY	16.2	
**Mean ± SEM**		6.3	27.0 ± 3.2		7.0 ± 0.9	7.6 ± 0.7
**Range**		4-12	10.8-52.6		0.4-9.6	5.0-9.7
**Cooled-shipped 48h**	9	6	22.5	SC	4.4	16.0
			24.5	EY	6.1	
	10	6	21.5	SC	0.4	14.0
			21.9	EY	0.9	
	11	n/a	23.5	SC	6.8	15.0
			23.8	EY	3.6	
	12	6	15.2	SC	4.7	17.0

			16.9	EY	5.0	
	13	6	17.3	SC	14.2	16.0
			19.4	EY	8.8	
	14	8	30.2	SC	5.8	15.0
			34.3	EY	6.7	
**Mean ± SEM**		6.4	22.6 ± 1.6		5.6 ± 1.0	15.5 ± 0.4
**Range**		6-8	15.2-34.3		0.4-14.2	14-17

Table S3. Donkey age, tail of epididymis weight, total sperm recovery harvested and temperature of the container at opening. Epididymides were shipped in passive cooling semen containers. Donkeys 1-20 were processed after arrival (**Cooled-shipped 24h**), whereas donkeys 21-36 were processed 24 h later (**Cooled-shipped 48 h**).

Cooled-shipped 24h						Cooled-shipped 48 h					
**Donkey**	**Age (yrs.)**	**Weight (g)**	**Extender**	**Total sperm ($x10^9$)**	**Temperature (°C)**	**Donkey**	**Age (yrs.)**	**Weight (g)**	**Extender**	**Total sperm ($x10^9$)**	**Temperature (°C)**
1	8	39.2	SC	11.6	7	21	11	23.9	SC	8.0	20
		40.2	EY	1.4				25.0	EY	11.6	
2	5	28.2	SC	8.6	7	22	11	27.6	SC	3.4	19
		28.2	EY	6.9				28.0	EY	4.4	
3	7	27.3	SC	9.3	7	23	10	29.2	SC	3.8	19
		27.0	EY	10.0				31.5	EY	11.2	
4	7	29.9	SC	4.5	7	24	11	25.2	SC	6.6	19
		31.8	EY	10				24.1	EY	8.6	
5	7	31.4	SC	5.7	8	25	6	22.8	SC	4.6	19
		n/a	EY	4.4				24.9	EY	11.0	
6	7	27.7	SC	9.5	8	26	10	24.2	SC	13.0	17
		31.3	EY	7.0				23.0	EY	2.2	
7	12	29.0	SC	11.0	9	27	9	29.2	SC	11.0	17
		29.0	EY	5.9				29.8	EY	18.0	
8	10	24.1	SC	15.9	9	28	7	35.3	SC	13.0	17
		41.9	EY	18.0				34.4	EY	5.6	
9	12	29.3	SC	9.6	9	29	9	25.4	SC	9.0	15
		29.4	EY	9.3				27.8	EY	7.7	
10	11	28.7	SC	19.0	8	30	7	27.0	SC	9.7	14
		28.4	EY	11.0				24.4	EY	8.2	
11	n/a	28.5	SC	7.0	8	31	12	28.3	SC	6.2	14
		25.9	EY	7.0				26.3	EY	10	
12	6	29.7	SC	10.0	8	32	17	27.6	SC	10	14
		28.2	EY	8.7				27.7	EY	6.9	
13	7	38.0	SC	12.0	10	33	9	26.0	SC	6.8	18
		45.0	EY	10.0				27.8	EY	8.8	
14	11	32.0	SC	8.9	11	34	3	25.1	SC	4.2	18
		32.5	EY	10.0				23.7	EY	8.0	

15	5	33.0	SC	7.2	10	35	8	28.8	SC	8.5	18
		36.2	EY	12.0				27.6	EY	9.4	
16	6	29.4	SC	9.2	11	36	15	26.5	SC	11.0	18
		32.0	EY	9.1				27.5	EY	8.5	
17	6	21.5	SC	1.4	13						
		22.0	EY	6.8							
18	8	27.2	SC	7.3	13						
		24.2	EY	4.7							
19	9	21.6	SC	6.7	13						
		20.8	EY	4.5							
20	4	22.4	SC	1.9	13						
		25.1	EY	0.4							
**Mean ± SEM**	7.8 ± 0.5	29.7 ± 0.9		8.3 ± 0.6	9.4 ± 0.5		9.7 ± 0.8	27.0 ± 0.5		8.4 ±0.6	17.2 ±0.5
**Range**	4-12	20.8-41.9		1.4-18	7-13		3-17	23.7-35.3		2.2-18	14-20
